# Early Life Nutrition and Mental Health: The Role of DNA Methylation

**DOI:** 10.3390/nu13093111

**Published:** 2021-09-04

**Authors:** Rola A Bekdash

**Affiliations:** Department of Biological Sciences, Rutgers University, Newark, NJ 07102, USA; rbekdash@newark.rutgers.edu

**Keywords:** brain, epigenetics, mental health, methyl donors, methylation, nutrition, one-carbon metabolism

## Abstract

Does the quality of our diet during early life impact our long-term mental health? Accumulating evidence suggests that nutrition interacts with our genes and that there is a strong association between the quality of diet and mental health throughout life. Environmental influences such as maternal diet during pregnancy or offspring diet have been shown to cause epigenetic changes during critical periods of development, such as chemical modifications of DNA or histones by methylation for the regulation of gene expression. One-carbon metabolism, which consists of the folate and methionine cycles, is influenced by the diet and generates S-Adenosylmethinoine (SAM), the main methyl donor for methylation reactions such as DNA and histone methylation. This review provides current knowledge on how the levels of one-carbon metabolism associated micronutrients such as choline, betaine, folate, methionine and B vitamins that play a role in brain function can impact our well-being and mental health across the lifespan. Micronutrients that act as methyl donors for SAM formation could affect global or gene methylation, altering gene expression and phenotype. Strategies should then be adopted to better understand how these nutrients work and their impact at different stages of development to provide individualized dietary recommendations for better mental health outcomes.

## 1. Introduction

Studies have shown an association between the quality of diet that we are exposed to during early life and mental health outcomes [1]. Nutritional status during early life, such as good diet or malnutrition, could shape our response and reaction to stress later on in life by epigenetic mechanisms [2]. This means that exposure in early life to environmental factors such as good or bad nutrients could alter our epigenome and cause changes in the expression of key genes in the brain by epigenetic mechanisms [3]. These epigenetic alterations induced by early environmental factors can be inherited across generations and leave a stable mark in the offspring [4]. According to the National Institute of Mental Health data in 2019, 51.5 million adults in the US have a mental health disorder [5]. The Centers for Disease Control and Prevention (CDC) reported that stress-related disorders such as depression, anxiety or behavioral disorders are common among children aged 3–17 [6]. These projected data indicate that mental health is a public health problem whose causes should be addressed in order to mitigate its outcomes. The quality of our diet and its impact on health play a role in this aspect.

Recent advances in genomics and epigenomics technologies and ongoing studies have increased our understanding of the effects of gene–nutrition interaction on brain health and disease. These effects are identified as changes in the expression of many genes and, in some cases, these changes are mediated by epigenetic mechanisms. In this manuscript, we will discuss how early-life nutrition is linked to mental health, with a focus on the effects of one-carbon metabolism associated micronutrients, which play a role in the formation of the methyl donor S-adenosylmethionine (SAM), on the etiology of stress-related disorders and neurodegenerative disorders.

## 2. Nutrients, Genes and the Epigenome

Emerging evidence indicates that our lifestyle and early-life nutrition could determine how susceptible or not we are to the development of diseases later in life. It is now recognized that epigenetic marks influenced by external factors such as nutrition are the link between our genes and our susceptibility to diseases. These external factors could modulate the epigenome by altering gene expression by epigenetic mechanisms such as DNA methylation [7]. Studies also are showing that diet during early life could affect neurodevelopment and then neurocognitive functions later in life [8]. Although more research is needed to demonstrate a causal relationship between the quality of nutrition during early life and neurocognitive performance, it is reasonable for health professionals and individuals to adopt strategies to optimize early-life nutrition for better mental health outcomes. Recent focus has been on the role of micronutrients that participate in the one-carbon metabolism in brain function. In this section, we will discuss this interplay between dietary micronutrients, one-carbon metabolism and DNA or histone methylation and how this interplay is crucial in gene regulation and in disease.

**Figure 1:**
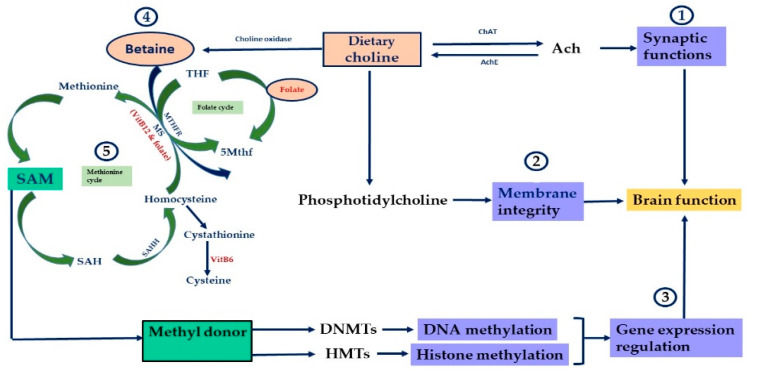
This figure shows how the micronutrients such as choline, betaine, folate, methionine and VitB12 are main players in the one-carbon metabolism and contribute to SAM formation, the main methyl donor for epigenetic mechanisms such as histone methylation and DNA methylation. (**1**) Choline is converted via choline acetyltransferase (ChAT) to acetylcholine (Ach), a major neurotransmitter that plays a role in cholinergic signaling. (**2**) Choline is also converted via several intermediary steps into phosphatidylcholine, a major structural component of cellular membranes. (**3**) Betaine, which is formed from choline via choline oxidase, contributes to the formation of SAM, a major methyl donor for key enzymes such as DNMTs, which catalyze DNA methylation, and HMTs, which catalyze histone methylation reactions. (**4**) Via its derivative betaine, choline also plays a role in the folate-mediated 1C metabolism. (**5**) Folate, VitB6 and VitB12 play important roles in SAM metabolism. After donating its methyl group, SAM is converted into S-adenosylhomocysteine (SAH), which is an inhibitor of methyltransferases. SAH is then hydrolyzed to homocysteine by S-adenosylhomocysteine hydrolase (SAHH). Homocysteine can be converted back into methionine via the transfer of a methyl group from 5Mthf by methionine synthase (MS), which requires the cofactors VitB12 and folate for its activity. Homocysteine can also be converted to cystathionine and then cysteine.

Epigenetics may be defined “as the study of any potentially stable and, ideally, heritable change in gene expression or cellular phenotype that occurs without changes in Watson-Crick base-pairing of DNA” [9]. Epigenetic mechanisms include covalent modification of DNA by methylation, post-translational modifications of histones and the role of non-coding RNAs such as microRNAs. These mechanisms are interrelated and intersect to impact chromatin structure and modulate gene expression [9]. DNA methylation is probably the best-characterized chemical modification of the chromatin that could be inherited across generations [4]. DNA methylation is a covalent modification that can be gene-specific or more global. It is catalyzed by a group of enzymes known as DNA methyltransferases (DNMTs). This modification happens on the cytosine residues of cytosine–phosphate–guanine (CpG) dinucleotides. CpGs are abundant in the promoter of most genes; the majority are unmethylated and are referred to as “CpG islands”. When these CpGs are abnormally methylated, they lock in the gene in a repressive or silent state, making it unlikely to be expressed. Some CG dinucleotides are located in intergenic or intragenic regions distant from the promoter. These CGs are called “Orphan CG” and are “CpG poor”. They could be methylated or unmethylated depending on the cellular physiological context and may play a role in gene expression regulation. Methylation of these islands in the gene promoter is often correlated with gene repression. Methylation of histones is catalyzed by a group of enzymes known as histone methyltransferases (HMTs or KMTs). Histone methylation happens at specific amino acid residues such as lysine or arginine on the N-terminal tail of histones and the effect of such modifications on gene expression depends on the respective amino acid that is methylated and how many amino acids are methylated. DNMTs or HMTs require the universal methyl donor S-adenosylmethionine (SAM) to methylate the DNA or histones [10], indicating that the availability of SAM is critical for cellular functions as it impacts gene expression regulation. It has been shown that there is a link between one-carbon metabolism and epigenetic mechanisms [11]. The one-carbon metabolism that consists of the folate and methionine cycles can generate SAM. SAM formation is dependent on the levels of cellular micronutrients that play a role in these cycles, such as folate, methionine, betaine, choline, VitB2, VitB6 and VitB12 [12,13] (Figure 1). These micronutrients can be derived from the diet, indicating that the quality of our diet across the lifespan will influence the one-carbon cycle and influence general health, including mental health [14,15,16,17,18,19].

Several studies demonstrated the role of dietary micronutrients during early life in altering gene expression and influencing health or disease phenotypes later in life by epigenetic mechanisms such as DNA methylation [20]. The alteration in the methylation patterns of CpGs in our genome is influenced by environmental cues or factors including nutrition and physical activity [21]. Could an epigenetic approach such as healthy nutritional supplementation mitigate or prevent negative effects on the epigenome? The role of micronutrients in altering global methylation or in altering gene-specific methylation has been studied in animal models and in select human studies. Data so far indicate that the alteration in the consumption of micronutrients such as folate, methionine, choline, betaine and B vitamins can alter phenotypes in a methylation-related mechanism by altering global or gene methylation during critical periods of brain development. For example, it has been long reported that neural tube defects, which indicate a failure of the closure of the neural tube during embryogenesis, are linked to a deficiency or a decrease in the levels of folic acid, choline and VitB12 in the diet or insufficient maternal intake of these micronutrients as supplements during pregnancy. The Food and Drug Administration action to authorize food fortification with folic acids and its recommendation for the intake of these micronutrients during pregnancy have decreased the prevalence of neural tube effects in the US [22,23]. These micronutrients are critical players in the one-carbon metabolism, are neuroprotective and are considered methyl donors since they contribute to the formation of SAM, a major substrate for almost all methylation reactions in mammals, including DNA and histone methylation [24]. It has been reported that the majority of methyl groups in one-carbon metabolism are derived from choline (60%), 20% from methionine and 10–20% from folate [25]. Normal levels of micronutrients have been shown to preserve the proper functioning of the methylation pathways that are essential for the neural tube closure in a cultured mouse embryo [26]. Similarly, neural tube defect mouse models *Axd mouse* (axial defects) that have abnormal levels of folic acid showed a reduction in the incidence of neural tube defects when given optimal levels of methionine, a precursor for the formation of SAM with a role in altering neurulation, during embryogenesis [27]. This suggests that alteration in the levels of SAM and hence alteration in methylation pathways are linked to the etiology of neural tube defects. Several human studies also implicated the role of prenatal choline and betaine intake in reducing the prevalence or the risk of neural tube defects in the newborn [28,29,30]. The role of maternal VitB12 deficiency in the etiology of neural tube defects is also reported [31,32,33,34,35]. At the global level, studies have shown an alteration in global methylation in the brains of Wistar rat offspring in response to changes in prenatal micronutrients in pregnant rats. This study demonstrated that the cortex of the adult offspring showed a state of hypermethylation in response to an imbalance in maternal micronutrients, such as an imbalance in the levels of folic acid and VitB12. Interestingly, prenatal supplementation of omega-3 fatty acid normalized methylation in the brains of the adult offspring [36]. This indicates that the intake of these micronutrients cannot be random but must be optimized to avoid unwanted activation of methylation pathways, which could alter the expression of key neuronal genes that should not be altered. Another study evaluated the impact of methyl donors during the perinatal period. Dams were fed with a high-fat diet (HF) during the perinatal period; then, the male and female offspring were fed postnatally (between 3 and 6 weeks of age) with a methyl-donor-supplemented diet (MDS). Perinatal HF and postnatal MDS diets altered the levels of folate metabolites and enzymes important for folate metabolism. The perinatal HF diet caused global DNA hypomethylation in the male offspring’s prefrontal cortex but not in the female offspring, suggesting that epigenetic regulation induced by early-life factors is sex-specific and brain-region-specific [37]. This hypomethylation was reversed to a level comparable to that of controls, with the postnatal MDS diet demonstrating the link between methyl donors and epigenetic phenomena such as methylation reactions.

A large-scale epigenome-wide DNA methylation analysis in the blood of participants was conducted to investigate the role of dietary folate and VitB12 intake in human health. This study examined around 485,512 CpGs in the whole blood of participants from Europe and North America. Data demonstrated differentially methylated regions (DMRs) and differentially methylation positions (DMPs) associated with folate or VitB12 intake. They identified 6 novel DMPs (markers for cell proliferation, tumor suppressor genes) and 74 DMRs (with relevance to immune function and stem cell proliferation) that are negatively associated with folate intake and 29 DMRs (most significant is the calcium-binding tyrosine phosphorylation-regulated that plays a role in fertility), of which 15 are negatively associated with VitB12 intake [38]. Although these findings are quite significant, replication of this large-scale study is needed before any definitive conclusion could be reached about the link between folate and VitB12 intake and the observed changes in methylation status in the blood of these participants.

Another human study examined the effects of maternal diet supplementation with folic acid during pregnancy on the cognitive functions of offspring. Pyrosequencing analysis showed significant changes in the methylation status of candidate genes related to brain development in the cord blood samples of newborns, demonstrating a link between maternal folate intake and offspring’s neurodevelopment. These genes are *LINE-1*, *TBM46*, *PEG3*, *APC2*, *OPCML*, *IGF2*, *BDNF*, *GRB10* and *GRIN3B*. Interestingly, folic acid intake during pregnancy correlated with a genome-wide decrease in methylation [39]. It has been argued that this study has limitations as other genes and CpGs were not analyzed and may have been affected by maternal folic acid supplementation during pregnancy. The changes in the methylation of the candidate genes in this study were detected in the cord blood, not in the brain.

A link between one-carbon-metabolism-associated micronutrients and changes in histone modifications was investigated in several studies [40,41]. For example, in a human embryonic stem cell (ESC) study, methionine deficiency decreased SAM levels, decreased the activation mark H3K4 trimethylation (H3K4me3) and resulted in defects in cellular self-renewal and ESC differentiation [42]. Prolonged methionine deprivation in human ESCs and induced pluripotent stem cells (ESCs/iPSCs) caused cellular apoptosis, demonstrating the role of methionine metabolism in the differentiation and maintenance of these cells [42]. Another study showed that in mice fed with a folate-deficient diet, H3K4 mono-methylation (H3K4me) increased in the liver, suggesting a reduction in lysine-specific demethylase (LSD1) [43]. Another study demonstrated that RIZ1 or KMT8, a tumor suppressor gene and a histone methyltransferase that catalyzes H3 lysine 9 (H3K9) methylation and causes transcriptional repression, was upregulated in mice fed with a methyl-balanced diet, whereas those fed with a methyl-imbalanced diet showed a reduction in hepatic SAM levels and the development of hepatic cancers [44]. Interestingly, the lysine-specific demethylase (LSD1) was found to be a folate-binding protein in nuclear extracts of HeLa cells, showing the link between folate and changes in methylation [45,46]. Other studies investigated the role of methyl donors in changes in histone arginine methylation. For example, Wistar rats fed with a diet high in methionine (HM) or a diet deficient in B vitamins (LV) or fed with both (HMLV) displayed an increase in homocysteine and S-adenosylhomocysteine (SAH) levels in the liver, and a reduction in H3 arginine 8 dimethylation (H3R8me2) in the brain [47]. Elevated levels of homocysteine have been linked to SAM deficiency or low SAM levels. In this study, mice fed with a diet for 12 weeks to induce hyperhomocysteinemia showed increased Enhancer of Zeste Homolog 2 (EZH2) expression, which catalyzes the trimethylation of H3 at lysine 27, with its associated mark H3K27me3 along the cystic fibrosis transmembrane conductance regulator (CFTR) promoter in the mouse liver, resulting in a decrease in *Cftr* gene expression. This indicates a correlation between methylation, EZH2 expression and the inhibition of *Cftr* expression in response to modulation in the mouse diet that affects the levels of homocysteine [48].

The role of prenatal and postnatal choline supplementation in several studies has been shown to exert positive effects on brain function and impact neural migration, differentiation and survival [49,50,51,52]. In the context of histone modifications, choline deficiency altered hippocampal development in the fetal brain and in cultured NPCs by causing gene-specific DNA methylation and an alteration in the chromatin landscape. In vivo studies showed that choline deficiency at embryonic day E17 resulted in a decrease in the expression of G9a histone methyltransferase and its associated marks, H3K9me1 and H3K9me2, in the subventricular zone and the ventricular zone of the hippocampus, with no changes in the global levels of these histone marks in the whole mouse fetal brain. These histone modifications correlated with a decrease in the binding of the repressor element 1-silencing transcription factor (REST) on the repressor element 1 (RE1) site of the calbindin gene (*Calb1*) promoter and an increase in *Calb1* expression in neural progenitor cells (NPCs), both in vivo and in vitro. Choline-induced changes in histone marks created an environment that is conducive to transcription upstream of the RE1 site of this gene [53]. It has been suggested that there is a correlative relationship between DNA methylation and the predominance of the methylated repressive mark H3K9 in causing gene repression [54]. Additionally, choline deficiency increased the methylation of one CpG site along the *Calb1* gene promoter in cultured NPCs, with no changes in total methylation of the CpG island of this gene [53]. Collectively, these data suggest that an alteration in choline levels during fetal life may have altered hippocampal neurogenesis by causing epigenetic changes in the fetal brain. Table 1 summarizes select studies conducted in humans and in animal models that demonstrated a link between one-carbon-metabolism-associated micronutrients and changes in global, gene or histone methylation.

Carcinogenesis is not only due to genetic factors but also to epigenetic events that are induced by environmental factors. The changes in global and gene methylation have been demonstrated in different types of cancer. More specifically, a state of global hypomethylation and a state of hypermethylation of specific genes such as tumor suppressor genes have been reported [64]. Studies have reported a link between the role of dietary methyl donors such as folate, methionine, choline, betaine and B vitamins in modifying global DNA or gene methylation and the development of different types of cancers [65]. This indicates that the intake of these micronutrients should be optimized and regulated.

Is there a link between the quality of our diet during early life and mental health across the lifespan? In the following sections of this manuscript, we will reveal that this relationship does exist and that this intricate relationship is modulated during critical periods of brain development and plasticity. Could we then use this knowledge to adopt a nutraceutical approach early in life coupled with physical activity to mitigate the negative effects of stress-related disorders and age-related cognitive dysfunctions that lead to neurodegenerative diseases later in life?

## 3. Early-Life Nutrition and Mental Health

There is a complex relationship between nutrition, genes and the brain, suggesting that an optimal energy status derived from the diet and regular physical activity can impact brain health, including mood and cognitive functions, across the lifespan. This impact of environmental influences such as nutrition or diet on brain health is explained by changes in gene expression, which could be dynamic, reversible, stable or even heritable across generations and contribute to phenotypic plasticity through epigenetic programming. Changes in gene expression are regulated by epigenetic mechanisms. In this section, the focus will be on the role of nutrition–gene interaction in mental health and well-being.

We are consistently subjected every day to stressors. We are quite different in our ability to cope with these stressors and whether these stressors could interfere with our daily work productivity and our state of well-being. Studies have demonstrated that several environmental factors, such as social, economic, cultural and nutritional, have an impact on our resilience in dealing with stressors and on our quality of life [66,67]. How much these epigenetic mechanisms are linked to the quality of diet is still not clear and should be further investigated.

Mental health disorders represent a global burden on societies, with economic costs and a reduction in health systems’ capacity to deal with the surge in mental illness and cognitive decline with aging. Our susceptibility to the development of stress-related disorders is linked to the type and duration of stressors and to the developmental period during which we are exposed to these stressors [68,69,70,71]. Nutrition or diet that we are exposed to in our lifetime have been implicated in the pathology of behavior-related problems, mood problems or mental illness, indicating that nutrition-based strategies and good lifestyle during early life could reduce the negative effects of mental disorders during adulthood [72]. Nutrition has recently emerged as a major factor in altering brain plasticity and function as there is an association between poor diet during early life and increased risk of developing mental disorders or cognitive impairments later in life [73,74,75,76]. Although it is very difficult to prove a causal relationship between the quality of dietary components or nutrition and mental health, data suggest that optimal controlled levels of micronutrients that participate in the one-carbon cycle may have beneficial effects on the brain and may have preventive and therapeutic outcomes [1]. For example, VitB12 deficiency can cause depression, reduced memory and psychosis [32,77,78]. Folic acid deficiency during critical periods of brain development, such as prenatal or during infancy, has negative effects on neurodevelopment and increases offspring’s risk of developing mental disorders during adulthood [35,79,80]. A placebo-controlled trial in children with ADHD who were not taking medications showed that the supplementation of a broad spectrum of micronutrients had beneficial effects on the psychological well-being of these children [81].

Although several diseases are caused by genetic factors, i.e., by alterations in the DNA sequence, a large number of diseases are now influenced by environmental factors that cause chemical modifications of the DNA by epigenetic mechanisms without altering its sequence [82]. The effects of environmental factors on the epigenome are more prominent and could shape adult phenotypes when the exposure to these factors happens early in life during critical periods of development, such as prenatal, postnatal, childhood and early adulthood [83]. Environmental cues such as diet or early-life nutrition could interact with neuronal genes and send their signals to epigenetic enzymes to epigenetically alter gene expression, leading to individual differences in behavior, cognitive functions and mental health later in life [84]. In some cases, the effects of environmental factors on the epigenome and alterations in gene expression and phenotypes could be transgenerational [3,4,85]. Some of the earliest human studies that showed the impact of early-life malnutrition on the health of offspring later in life were the Dutch Hunger Winter famine (1944–1945) [86,87,88] and the Chinese Great Leap Forward famine (1959–1961) [89]. For example, individuals who were prenatally exposed to malnutrition during the Dutch Hunger Winter famine showed hypomethylation in the imprinted *IGF2* gene. This epigenetic methylation mark persisted in these individuals throughout life, showing the profound effects of early-life exposure on the epigenome [90]. Another study showed changes in the methylation status of specific genes in individuals prenatally exposed to famine that were sex-specific and specific to the time of exposure during the gestational period [91,92]. This change in methylation status indicates that diet has an impact on our genes, but how could we protect our health from this? The viable yellow Agouti mouse study proved that supplementation of the maternal diet with methyl donors (folate, VitB12, choline and betaine) could reverse the phenotype of offspring by altering the methylation of a transposable element, the intracisternal A particle (IAP) located upstream of the *Agouti* gene, shifting the phenotype of offspring towards a brown color, with a reduced possibility of developing cancer, diabetes and obesity [59]. This clearly indicates that the intake of methyl donors as supplements should not be abused and the levels and duration of intake should be controlled to prevent unwanted changes in gene expression regulation.

In the context of brain function, several factors such as micronutrients and early-life nutrition play a role in programming long-term health, including brain health [66]. Although several macronutrients, micronutrients and the type of diet are involved, the focus in this section is on the effects of those micronutrients that play a role in one-carbon metabolism and alter gene expression by epigenetic mechanisms. Choline, a methyl donor, has been recently implicated in the effects of substance abuse such as alcohol abuse, a strong environmental factor, on the brain and in the programming of the hypothalamic–pituitary–adrenal (HPA) stress axis function by epigenetic mechanisms. Prenatal choline supplementation altered the methylation of the histone activation mark H3K4me3 and the histone repressive mark H3K9me2 in β-endorphin-producing proopiomelanocortin (POMC) neurons in the arcuate nucleus of the hypothalamus of exposed offspring. It also decreased the methylation pattern along the *Pomc* gene, elevated its mRNA expression in the hypothalamus and decreased the levels of the stress hormones adrenocorticotropic hormone (ACTH) and corticosterone in adult offspring. These data suggest a potential role of choline in attenuating some of the adverse effects of prenatal alcohol exposure, such as increased stress reactivity in the adult stage [58]. A human study also showed that maternal choline supplementation attenuated the HPA stress axis reactivity in offspring. In particular, it altered the methylation status along the promoter of some cortisol-regulating genes, including the corticotropin-releasing hormone (*Crh*) gene and glucocorticoid gene Nr3c1 in the placenta and in the cord blood, resulting in lower circulating levels of the stress hormone corticosterone in cord plasma [55].

Another human study investigated the effects of methyl donor intake, such as methionine, choline, betaine and VitB2, VitB6 and VitB12, during the early postnatal period (first 2–3 years) on changes in global methylation in the buccal cells of children at age 4. Although DNA methylation levels were higher in males than females in response to this intake, no association was detected between the intake of those micronutrients that contribute to the one-carbon metabolism and changes in global methylation [56]. The methionine cycle and folate cycle are the two main cycles in the one-carbon metabolism, with a contribution of VitB12 and folic acid in the normal functioning of these cycles (Figure 1). Abnormality in the functioning of these two cycles due to a deficiency in important enzymes, vitamins or micronutrients such as folate and/or VitB12 could curtail the formation of the methyl donor SAM and has been linked to mental illness, psychiatric disorders and neurological disorders due to alterations in neuronal gene expression [93,94,95]. As stated previously, a methyl donor deficiency will reduce the levels of SAM and elevate the levels of homocysteine. A study reported an inverse correlation and relationship between elevated levels of maternal plasma homocysteine due to methyl donor deficiency at preconception and psychomotor and cognitive development scores in children at 4 months and 6 years of age postnatally [96]. Another human study investigated the effects of one-carbon micronutrient deficiency, such as folate, betaine and 5′-methyltetrahydrofolate (5Mthf), in prenatal maternal blood on neurodevelopment and increased risk of autism in children after birth. Gene expression analysis of maternal blood revealed changes in the expression of immune, apoptotic, epigenetic and development-related genes, suggesting an association between the deficiency in one-carbon nutrients and neurodevelopmental delay and the development of autism in children [97]. Another neurodevelopmental disorder, Rett Syndrome, is considered one of the main causes of mental retardation in girls and is due to a deficiency in part of the epigenetic machinery, such as the methyl–CpG binding protein 2 (MeCP2) [98]. Although there is a scarcity of data on the impact of one-carbon nutrients on this progressive disorder, animal studies conducted in mice or rats proved that these nutrients could improve or mitigate the symptoms. For example, supplementation of dietary choline, which is known to be important in brain function and development, into an MeCP2-conditional knockout mouse improved its motor coordination and reduced anxiety-like behavior, leading to behavioral changes. These behavioral changes induced by choline were also associated with morphological changes in cortical neurons, seen as an increase in the soma size of these neurons, an increase in the complexity of their dendritic spines and an increase in the expression of synaptic proteins, suggesting an improvement in neurotransmission [99]. A limited human study conducted in four female Rett patients demonstrated a lowered level of 5-methyltetrahydrofolate (5-Mthf) in the CSF, with a reduction in total folate-binding capacity to folate-binding proteins (FBPs). Supplementation of folinic acid improved the levels of 5-Mthf in the CSF of these patients [57].

Studies using animal models such as rats or mice provided useful data on the effects of early-life nutrition, such as the availability of one-carbon nutrients, on health and disease later in life. For example, pregnant rats fed with a diet that was low in methyl donors resulted in a state of hypomethylation in the offspring, with changes in histone marks such as increased H3K9 acetylation (H3K9Ace), along the hepatic glucocorticoid receptor gene exon (GR), with a decrease in DNMT1 expression in adult offspring. These epigenetic changes induced by restrictions in the maternal diet were reversed or reduced by maternal supplementation of the diet with folic acid [60,100]. The paternal diet also has been shown to cause epigenetic programming of offspring. Elevated paternal dietary methyl-donor intake in a mouse model impaired cognitive function in the offspring by showing an impairment in memory and learning, behavioral changes that were associated with changes in the expression of the methionine adenosyltransferase (*Mat2a*), which led to the formation of SAM, and changes in the methylation status of the BK channel subunit *Kcnmb2* gene promoter [62]. A paternal diet that is deficient in methyl donors such as folate, methionine and choline (FMCD diet) in F0 male mice resulted in behavioral changes in the F1 generation. These changes were associated with changes in the expression of memory-related genes such as *CamK2α* and Protein Phosphatase *PP1* and changes in the methylation of PP1 promoter in the hippocampus. These findings indicate that the paternal intake of methyl donors in the diet could cause behavioral changes and gene expression changes in the offspring by epigenetic mechanisms such as methylation changes [63]. Another study demonstrated neurochemical changes in different brain regions and behavioral changes in rats who were fed for 11 weeks with a diet deficient in methyl donors such as VitB2, B9, B12 and choline. Methyl-donor deficiency resulted in reduced levels of plasma corticosterone and elevated levels of plasma homocysteine, with reduced concentrations of 3,4-Dihydroxyphenylacetic acid (DIPAC) (a metabolite of dopamine) and 5-Hydroxyindolacetic acid (5HIAA) (a major metabolite of serotonin) in the striatum and the hypothalamus. Subjecting these rats to unpredictable chronic mild stress (UCMS) amplified the effects of the methyl-donor deficiency, as demonstrated by the open field and forced swim tests [101]. This suggests an association between methyl-donor deficiency and stress-related disorders.

## 4. Micronutrients and Neurodegenerative Disorders

Diet is an important environmental factor that plays a critical role in the growth of cells and has an influence on cognitive functions and brain health [102]. Adequate intake of methyl-donor nutrients that contribute to SAM formation, which is necessary for epigenetic mechanisms, plays a role in proper brain functioning [103]. Epigenetic mechanisms have been linked to cognitive decline with aging and the etiology of neurodegenerative disorders [104,105,106,107,108]. Several studies conducted in animal models and in humans reported a strong association between suboptimal nutrition during early life, such as during the fetal stage or postnatal period, and the predisposition to neurodegeneration or decline in cognitive functions later in life [109]. In this section, a summary of research findings is presented to show how an alteration in the levels of specific micronutrients, one-carbon nutrients, during early life can cause epigenetic changes in the expression of key neuronal genes by changes in DNA methylation or histone modifications and alter cognitive functions later in life.

The link between nutrition and mental health has been extensively studied. For example, VitB12 and omega-3 fatty acids have been shown to have positive effects on cognitive functions and mental health but the underlying mechanisms of how these nutrients work still need to be established. VitB12 is a micronutrient that plays a role in brain function. It is associated with one-carbon metabolism as it acts as a cofactor for methionine synthase (MS), which converts homocysteine to methionine and contributes to the formation of SAM, a major methyl donor for epigenetic mechanisms such as DNA and histone methylation [110] (Figure 1). An elevation of homocysteine due to ViB12 deficiency has been shown to elevate the levels of reactive oxygen species (ROS), cause neuronal DNA damage and impact brain function [111,112]. A low intake or deficiency of VitB12 during pregnancy influences the cognitive functions of offspring at 9 years of age [113]. A study conducted in pregnant Wistar rats showed that the supplementation of the maternal diet with VitB12 and omega-3 fatty acids increased the levels of brain-derived neurotrophic factor (BDNF) in the hippocampus of offspring at 3 months of age, with an improvement in their spatial memory performance, as shown by the radial arm maze test, suggesting a role of these nutrients in promoting cognitive functions during adulthood [114].

In the context of stress, a study investigated the effects of maternal diet supplementation with micronutrients such as methionine and B vitamins during the early-life stress period (ES) in C57Bl/6 mice on the effects of ES on the adult offspring. Maternal diet supplementation with micronutrients normalized the levels of methionine in the plasma and in the hippocampus and improved offspring performance in the Morris water maze test. It also normalized the ES-induced hyperactivation of the stress axis in the offspring, with no observed changes in the expression of glucocorticoid receptor (GR) or changes in global DNA methylation [61]. A human study assessed the effects of early-life exposure to Chinese famine on adult cognitive functions later in life. This study, which was conducted in 1162 adults, demonstrated using several tests a strong association between early-life exposure to famine and an increased risk of cognitive decline during adulthood [115].

## 5. Optimization of Intake of One-Carbon-Metabolism-Associated Micronutrients

Effective and safe strategies should be developed to ensure proper intake of micronutrients at different stages of development, establish dose–response relationships in different populations and consider other factors for better mental health outcomes [116]. When data are available, dietary recommendations could then be applied during early life or later in life to prevent or mitigate cognitive decline with aging. There are mixed results related to the effects of one-carbon-metabolism-associated micronutrients on cognition in human studies compared to animal studies. This fact necessitates a more holistic approach to how studies are conducted and what each study is looking for. The public should be educated on the adverse effects of excessive intake of these micronutrients as supplements, indicating that their intake should be discussed with a health professional by taking into consideration the medical history and the genetic profile of each individual. We should then aim to have a healthy diet and healthy lifestyle for good mental health outcomes. Since the demand for one-carbon-associated micronutrient intake is high during pregnancy, the development of a one-carbon metabolism requirement model is needed for pregnant women to support optimal maternal and offspring health outcomes [117].

Adults in specific age groups in the US consume less of what is needed in terms of micronutrients [118] and adults of all ages consume less than the 2020–2025 Dietary Guidelines recommended portions of most healthy groups [119]. A similar finding was reported in older adults in Europe, indicating that this problem is global [120]. Healthy food group servings that provide adequate levels of energy in older adults were recommended by the US dietary guidelines in different types of diets, such as the Mediterranean and vegetarian diet [119]. The estimated average requirement (EAR) of specific micronutrients including VitB12 and choline was reported for different age groups [121]. There must be a national nutrition strategy that could be implemented to promote healthy aging. This means a governmental commitment to funding nutrition-related research and training sessions or workshops at schools and in educational institutions related to this matter in order to promote public awareness about the effects of the quality of early nutrition on brain health and well-being. We also need to take advantage of technology such as artificial intelligence in an effective way in order to collect data from routine screening from diverse populations in different age groups related to food intake, lifestyle and frequency of exercise per day, in order to devise personalized dietary/supplement interventions and weight management techniques.

## 6. Limitations and Future Considerations

It is becoming increasingly evident that there is an association between the quality of nutrients during early life and mental health outcomes. Mental well-being is a core element for longevity, increased productivity, reduced medical cost burden and improving the quality of life. Although mental disorders are complex and some are multigenic due to genetic factors, studies have demonstrated that environmental factors such as stress, quality of diet and frequency and duration of physical activity could improve or worsen the symptoms of these disorders. Despite this, there is no causative relationship but a correlation between the implementation of a healthy nutritional strategy in early life and good mental functioning throughout life. The reasons behind this could be related to several factors, such as the unwillingness of individuals to consistently share information with their physicians about their diet, a lack of institutional commitment to educating the public about the beneficial effects of healthy nutrients on long-term mental health and insufficient governmental funds to support research related to this topic. Limitations in this field of gene–nutrition interaction are also related to inconsistencies in studies that are conducted in animal models, which impose challenges in extrapolating these findings and implementing them in humans. Humans are quite complex, and it is difficult to follow humans’ diet throughout the lifespan, as we do in animal models, to devise long-term solutions and accurate conclusions. Other limitations in this field could be attributed to interindividual differences in response to dietary intake that should be considered by health professionals, such as genetic variations, differences in health status and dietary preferences and exposure to different environmental factors or influences. Future research should continue to identify the mechanism of micronutrients and how they alter genes in different metabolic pathways in the human body and in the brain. This knowledge has potential applications to improve human health outcomes and possibly delay or mitigate the symptoms of diseases.

Technology has advanced rapidly in recent years, enabling us to invest in it for good purposes to improve the quality of life. We now have the capability to use data analytics to understand human behavior and preferences, in order to tailor services in any market to match consumers’ demand, whether in retail, education or in the movie industry. Similarly, health professionals could collect data from patients and, with their consent, on their eating habits early in life and at different stages of life. The collection of data in such a marketplace would enable physicians to identify patterns that enable them to provide individualized strategies on dietary recommendations that are affordable and suit different needs, taking into consideration an individual’s age and gender, in order to prevent, manage or delay specific medical conditions. Although we cannot exclude genetic factors that can affect individual’s mental health, understanding how the epigenome is different from the genome and how nutrients alter genes is essential. This knowledge would help physicians and individuals to adopt and implement a nutritional strategy early in life to improve the quality of life and prevent the development of other diseases such as cardiovascular disease, diabetes and obesity later in life. As stated earlier, the use of those micronutrients that play a role in the one-carbon metabolism should be monitored and regulated by health professionals. Such regulation will prevent the random and uncontrolled use of these micronutrients by individuals and eliminate unwanted and unexpected health side effects. The challenge would then be to motivate people to adopt and implement a recommended healthy diet and continuously educate the public about the effects of the quality of nutrients on health and well-being.

## 7. Conclusions

The role of early-life nutrition in brain health has been extensively studied and there is a strong relationship between early-life nutrition, susceptibility to stress, mental health and well-being across the lifespan. Micronutrients such as choline, betaine, methionine, folate and B vitamins are derived from our diet and could be found as supplements. They have been shown to alter methylation pathways since they contribute to the functionality of the one-carbon metabolism and to the formation of the universal donor, SAM. SAM donates a methyl group to enzymes that methylate genes or histones and alter gene expression. Studies conducted mostly in animal models have indicated the critical role of these micronutrients in good mental health during early life or during critical periods of development. There is no doubt that this relationship between our genes and our diet is existent but complex. Although genetic factors play an important role in our disposition to several diseases, environmental factors, such as controlled diet and regular physical activity, have been shown to improve the quality of life, increase longevity and reduce our susceptibility to diseases later in life. Medical practices should then adopt different strategies in understanding the etiology of mental-health-related diseases by applying epigenetically driven approaches to understand how a specific diet or supplementation of micronutrients in a regulated manner during early life interact with neuronal genes or even metabolic genes and health outcomes. This could drive more effective personalized dietary recommendations early on for individuals based on their genetic and epigenetic profiles. More studies are needed to better understand the impact of micronutrients during early life on our epigenetic profile and on our mental health and well-being throughout life.

## Figures and Tables

**Table 1:** This is a table that summarizes studies conducted in humans and animal models that showed a link between one-carbon-metabolism-associated micronutrients and methylation.

Description	Outcomes	References
Prenatal choline and betaine intake in humans	Reduction in NTD risk	[26,28,29]
Maternal choline supplementation in humans	Attenuation of the stress axis in offspring Alteration in the methylation of *Crh* and *Nr3c1* in placenta and in cord blood with a decrease in corticosterone levels in cord plasma (*n* = 29)	[55]
Maternal diet supplementation with folic acid during pregnancy	Changes in methylation of candidate genes related to brain development in blood samples of newborns (*n* = 86) with a genome-wide decrease in methylation	[39]
Maternal supplementation with folic acid in humans	Genome-wide decrease in methylation, alteration in the methylation status of genes in the offspring (*n* = 5841)	[38]
Methyl donor intake during early postnatal period (2–3 years)	Higher methylation in buccal cells of males compared to females in children at age 4 with no proven association between methyl donor intake and changes in global methylation (*n* = 73)	[56]
Supplementation of folinic acid in female Rett patients	Improved levels of 5-MTHF in CSF of female patients (*n* = 4)	[57]
Prenatal choline supplementation in alcohol-exposed pregnant rats	Increase in H3K4me3, decrease in H3K9me2 in β-endorphin-producing neurons in the hypothalamus of exposed offspring with a decrease in *Pomc* gene methylation and decrease in ACTH and corticosterone levels in the blood	[58]
Choline deficiency at E17 in mice	Decrease in G9a, decrease in H3K9me1 and H3K9me2 in the SVZ and ventricular zone in mice hippocampus with no changes in global levels of histone marks in the mouse fetal brain. Decrease in binding of REST on *Calb1* gene promoter with an increase in Calb1 expression in NPC	[53]
Methionine intake in Axd mutant mice	Reduction in NTD	[27]
Imbalanced levels of VitB12 and folic acid in pregnant Wistar rats	Hypermethylation in the cortex of adult offspring	[36]
Methyl-balanced diet in mice	Increase in KMT8 expression and cancer prevention in liver	[44]
Folate deficient diet in mice	Increase in H3K4me in liver	[43]
Wistar rats fed with HM, LV or HMLV	Increase in homocysteine and SAM in the liver, and decrease in H3R8me2 in the brain	[47]
Mice-induced hyperhomocysteinemia	Increase in EZH2, increase in H3K27me3 along *Cftr* gene promoter and decrease in *Cftr* expression	[48]
Prolonged methionine deprivation in ESCs/iPSCs	Cellular apoptosis	[42]
Methionine deficiency in hESCs	Decrease is SAM levels, decrease in H3K4me3 Impacted differentiation of ESC	[42]
Maternal diet supplementation with methyl donors in viable yellow Agouti mice	Alteration in the methylation status of IAP and *Agouti* gene expression and shifting phenotype toward the brown color	[59]
Pregnant rats fed with diet low in methyl donors	Hypermethylation, increase in H3K9Ace along hepatic GR gene promoter and decrease in DNMT1 expression in the offspring	[60]
Maternal diet supplementation with methionine and B vitamins in ES mice	Normalization in methionine levels in plasma and hippocampus and stress axis in offspring with no changes in global DNA methylation	[61]
Elevated paternal dietary intake of methyl donors in mice	Changes in *Mat2a* expression and changes in methylation of Kcnmb2 gene promoter in offspring	[62]
Paternal diet deficient in methyl donors in mice (FMCD diet)	Changes in methylation of PP1 gene promoter in the hippocampus of offspring	[63]

Abbreviations: 5-methyltetrathydrofolate (5-MTHF), neural tube defect (NTD), cerebrospinal fluid (CSF), subventricular zone (SVZ), repressor element-1 silencing transcription factor (REST), high in methionine (HM), deficient in B vitamins (LV), fed with both (HMLV), proopiomelanocortin (Pomc), corticotropin-releasing hormone (Crh), cystic fibrosis transmembrane conductance regulator (Cftr), adrenocorticotropin hormone (ACTH), neural progenitor cell (NPC), embryonic day 17 (E17), embryonic stem cell (ESC), induced pluripotent stem cell (iPSC), intracisternal A particle (IAP), glucocorticoid receptor (GR), DNA methyltransferase (DNMT), Enhancer of Zeste Homolog 2 (EZH2), protein phosphatase 1 (PP1), acetylation of histone 3 at lysine 9 (H3k9Ace), trimethylation of histone 3 at lysine 4 (H3K4me3), trimethylation of histone 3 at lysine 27 (H3K27me3), dimethylation of histone 3 at arginine 8 (H3R8me2), axial defect (Axd), BK channel subunit (Kcnmb2), dimethylation of histone 3 at lysine 9 (H3k9me2), monomethylation of histone H3 at lysine 9 (H3K9me1), methionine adenosyltransferase (Mat2), calbindin (calb1), glucocorticoid gene (Nr3c1).

## Data Availability

Not applicable.
